# Focal neocortical lesions impair distant neuronal information processing

**DOI:** 10.1113/JP277717

**Published:** 2019-07-25

**Authors:** Anders Wahlbom, Jonas M. D. Enander, Fredrik Bengtsson, Henrik Jörntell

**Affiliations:** ^1^ Neural Basis of Sensorimotor Control Department of Experimental Medical Science BMC F10 Tornavägen 10 SE‐221 84 Lund Sweden

**Keywords:** Neocortex, stroke, neuron, information processing, tactile, touch, spreading depression, photothrombosis, somatosensory cortex, parietal cortex

## Abstract

**Key points:**

- Parts of the fields of neuroscience and neurology consider the neocortex to be a functionally parcelled structure.
- Viewed through such a conceptual filter, there are multiple clinical observations after localized stroke lesions that seem paradoxical.
- We tested the effect that localized stroke‐like lesions have on neuronal information processing in a part of the neocortex that is distant to the lesion using animal experiments.
- We find that the distant lesion degrades the quality of neuronal information processing of tactile input patterns in primary somatosensory cortex.
- The findings suggest that even the processing of primary sensory information depends on an intact neocortical network, with the implication that all neocortical processing may rely on widespread interactions across large parts of the cortex.

**Abstract:**

Recent clinical studies report a surprisingly weak relationship between the location of cortical brain lesions and the resulting functional deficits. From a neuroscience point of view, such findings raise questions as to what extent functional localization applies in the neocortex and to what extent the functions of different regions depend on the integrity of others. Here we provide an in‐depth analysis of the changes in the function of the neocortical neuronal networks after distant focal stroke‐like lesions in the anaesthetized rat. Using a recently introduced high resolution analysis of neuronal information processing, consisting of pre‐set spatiotemporal patterns of tactile afferent activation against which the neuronal decoding performance can be quantified, we found that stroke‐like lesions in distant parts of the cortex significantly degraded the decoding performance of individual neocortical neurons in the primary somatosensory cortex (decoding performance decreased from 30.9% to 24.2% for *n* = 22 neurons, Wilcoxon signed rank test, *P* = 0.028). This degrading effect was not due to changes in the firing frequency of the neuron (Wilcoxon signed rank test, *P* = 0.499) and was stronger the higher the decoding performance of the neuron, indicating a specific impact on the information processing capacity in the cortex. These findings suggest that even primary sensory processing depends on widely distributed cortical networks and could explain observations of focal stroke lesions affecting a large range of functions.

## Key points

- Parts of the fields of neuroscience and neurology consider the neocortex to be a functionally parcelled structure.
- Viewed through such a conceptual filter, there are multiple clinical observations after localized stroke lesions that seem paradoxical.
- We tested the effect that localized stroke‐like lesions have on neuronal information processing in a part of the neocortex that is distant to the lesion using animal experiments.
- We find that the distant lesion degrades the quality of neuronal information processing of tactile input patterns in primary somatosensory cortex.
- The findings suggest that even the processing of primary sensory information depends on an intact neocortical network, with the implication that all neocortical processing may rely on widespread interactions across large parts of the cortex.

## Introduction

An understanding of how the information processing is organized in the neocortex is crucial for neuroscience, neurology and brain‐inspired computational strategies. The idea of functional localization in the brain, i.e. that each region of the cortex carries out a specific function, originates from the 19th century (Broca, 1861; Penfield & Boldrey, 1937) but is still pervasive in neuroscience. Numerous modern experimental studies of the human brain report compatible results (Maldjian *et al*. 1999; Desmurget & Sirigu, 2015) although it is not clear how far‐reaching the local specialization is. Neurology has similarly adopted the view and, consequently, many current treatment strategies are based on the tenet that there is a straightforward relationship between the location of the lesion and the overt symptoms found by the clinician: ‘A cornerstone of clinical neurology is that focal brain injury causes specific behavioural symptoms or syndromes that reflect the functional specialization of different brain modules’ (Corbetta *et al*. 2015). However, there is an increasing notion that the fundamental aspects of this tenet may not be correct (Bassetti *et al*. 1993; Connell *et al*. 2008; Corbetta *et al*. 2015; Sathian & Crosson, 2015), supported by striking observations that unilateral stroke affects tactile function bilaterally, regardless of the location of the stroke (Kim & Choi‐Kwon, 1996; Brasil‐Neto & de Lima, 2008).

An alternative view to functional localization would be that all brain functions to some extent depend on all parts of the brain. A local loss of brain tissue would then result in an impairment of most brain functions and the larger the loss, the larger the general impairment, similar to the interpretation of Goltz (1888), Phillips *et al*. (1984), Tyler & Malessa (2000) and Lashley (1929). Such a globalized view of brain function is supported by the fact that many localized cortical infarcts pass unnoticed (Wardlaw *et al*. 2013) as well as by some models of brain function (Tononi & Edelman, 1998; Engel *et al*. 2013).

A critical issue for the debate on localized *versus* globalized brain processing is to what extent the information processing in different cortical areas is dependent on that in the other areas. The issue has been hard to address, since it requires a more finely graded performance measure than the mere identification of the presence or absence of a response. Here we analyse the effects of distant localized cortical stroke‐like lesions using a gradable measure of the information processing in individual neurons in the primary somatosensory cortex (S1), based on the delivery of a highly reproducible set of spatiotemporal tactile afferent activation patterns to the second digit (Oddo *et al*. 2017; Genna *et al*. 2018). The main finding is a consistent reduction of the decoding performance in these neurons. The results indicate that even primary sensory processing depends on widely distributed circuitry in the brain and that focal stroke would therefore be expected to have widespread functional consequences.

## Methods

### Ethical approval

All animal experimental procedures in the present study were in accordance with institutional guidelines and were approved in advance by the Local Ethics Committee of Lund, Sweden (permit ID M118‐13). All experiments were performed using acute preparations under general anaesthesia. All experimental procedures comply with *The Journal*’s principles and guidelines on animal experimentation as defined in the editorial by Grundy (2015).

### Preparation

Adult Sprague–Dawley rats (*n* = 12, weight 304–385 g, of male sex; plus *n* = 6 rats of male sex weighing 309–380 g for sham experiments) were prepared and maintained under a ketamine/xylazine anaesthesia mixture. Prior to the induction the animal was sedated with isofluorane (3% mixed with air for 60–120 s). The anaesthesia was induced with an intraperitoneal injection (ketamine:xylazine concentration ratio of 15:1, initial dose approximately 60 mg/kg for the ketamine component), and further maintained with a continuous infusion through an intravenous catheter inserted into the right femoral vein (corresponding to approximately 5 mg/kg per hour for the ketamine component, end concentration ratio of 20:1 for ketamine:xylazine). The absence of withdrawal reflexes to noxious pinch to the hindpaw was used to characterize adequate anaesthesia until the brain was exposed. After that the level of anaesthesia was monitored with an electrocorticogram (ECoG) electrode and as well as continued absence of withdrawal reflexes. The ECoG was monitored for irregular occurrences of sleep spindles indicating deep sleep (Niedermeyer & da Silva, 2005).

Anaesthesia is known to affect the efficacy of synaptic transmission (Bengtsson & Jörntell, 2007) and therefore, by implication, the function of the brain. However, the physiological structure of the network, which reflects the learning history, and therefore, is a main determinant of brain information processing, is apparently not altered as the order of recruitment of a population of neurons to a given stimulus, or during spontaneous Up‐states, does not change with anaesthesia (Luczak & Bartho, 2012). Anaesthesia will moreover have the beneficial effect that there are no systematic variations in brain states preceding the onset of the stimulation (Wallach *et al*. 2016). In the awake state, it is difficult to know to what extent the recorded neural activity is induced by the afferent input or by internal brain activity, for example predictions or expectations made by the animal (Eskandar & Assad, 1999). Therefore, for the purpose of this study, we believe that the advantages of the anaesthesia outweigh its disadvantages.

In order to access the surface of the neocortex for recordings, a craniotomy (approximately 3 mm by 3 mm) was made at the location of the forepaw region of the primary somatosensory cortex (S1) (Paxinos & Watson, 2006). All recordings were made at depths between 0.44 and 1.25 mm. Note that we have previously reported that there is no relationship between recording depth and decoding of the tactile afferent inputs we used (Oddo *et al*. 2017). The ECoG electrode was placed at the rostral end of the craniotomy. A pool of cotton‐in‐agar was built and filled with warm (37°C) paraffin oil to prevent the exposed parts of the brain from drying. Another partial craniotomy (bone thinning) was made over the parietal/occipital region to allow the induction of stroke (see below).

### Recordings

Using two separate patch clamp recording electrodes, individual neurons were recorded extracellularly in the loose patch current clamp recording mode. Patch clamp pipettes were pulled from borosilicate glass capillaries to 10–30 MΩ using a Sutter Instruments (Novato, CA, USA) P‐97 horizontal puller. The composition of the electrolyte solution in the patch pipettes was (in mm): potassium gluconate (135), Hepes (10), KCl (6.0), Mg‐ATP (2), EGTA (10). The solution was titrated to 7.35–7.40 pH using 1 m KOH. During slow advancement of the recording electrode (approximately 0.2 µm/s) with an electrical stepping motor, all the skin stimulation sites were activated at one pulse per second to activate the neurons. Any neuron encountered was recorded if possible. All data were digitized at 100 kHz using CED 1401 mk2 hardware and Spike2 software (Cambridge Electronic Design (CED), Cambridge, UK). The depth from the surface of the brain was recorded. After the recording session the animals were killed with a lethal dose of pentobarbital.

The exposed part of the brain was inspected with a microscope during both insertion and extraction of the recording electrodes for detection of signs of deterioration of the state of the brain (i.e. bleeding on the cortical surface for example). The state of the rat was continuously evaluated based on skin tone, respiration rate and ECoG signal. Furthermore, the duration of the anaesthesia did not exceed 8 h.

### Stimulation

Four pairs of intracutaneous needle electrodes were inserted with inter‐needle distance of 2–3 mm into predetermined sites in the skin on the volar side of digit 2 of the left forepaw. These constituted the electrical interface to the tactile afferents, i.e. the same approach as reported in a previous study (Oddo *et al*. 2017). For each skin site the stimulation pulse was set to an intensity of 0.5 mA with a duration of 0.14 ms (DS3 Isolated Stimulator, Digitimer, Welwyn Garden City, UK), which is 2.5 times the threshold for activating tactile afferents (Rasmusson & Northgrave, 1997; Bengtsson *et al*. 2013) and therefore was well below the recruitment threshold for Aδ‐ and C‐fibres in the skin (Ekerot *et al*. 1987). Through this interface eight predefined spatiotemporal patterns of skin activation were delivered (the stimulation patterns are indicated as F5, S5, F10, S10, F20, S20, F∞ and S∞, and were exactly the same as in the paper of Oddo *et al*. 2017). These stimulus patterns lasted less than 350 ms (range 213–346 ms) and consecutive deliveries were separated by 1.8 s. The spatiotemporal patterns were presented repeatedly up to 100 times in a pseudo‐random order. The generation of the stimulation patterns has been described in detail previously (Oddo *et al*. 2017) and that paper also contains an extensive discussion on the advantages and limitations of this approach. Since each stimulation pattern was repeated 100 times, we could display the evoked responses as peristimulus time histograms (PSTHs). To generate a better representation of the evoked spike responses than provided by traditional PSTHs only, we transformed the spike times to a spike density function (i.e. a form of kernel density estimation (KDE)). For each neuron, the individual spike responses were grouped by stimulation pattern and the corresponding KDE for each group, or stimulation pattern, was calculated using the solution of Shimazaki & Shinomoto (2010). KDE provides a more accurate representation of the spike time data than the PSTH since it avoids the loss of information associated with binning.

### Induction of the photothrombotic lesions

As a model for stroke, we used a photothrombotic lesion in the neocortex of the right hemisphere. This approach has an advantage over temporary inactivation approaches in that it provides a robust lesion covering all neurons in the affected region, which moreover can be morphologically verified, and that the extent of the lesion/inactivation does not vary over time as the conditioned cortical responses were recorded. The location of the photothrombotic lesion was the centre of the right hemicranium, measured as half the distance between bregma and lambda, and half the distance between the mid‐line suture and the lateral bony ridge of the masseter muscle. The location was selected because it represented a cortical area with a reasonably large distance from the S1, was easily accessed and at the same time minimized the risk of interfering with major blood vessels during the bone thinning and the laser illumination. The photothrombotic lesion was induced according to the description of Shanina *et al*. (2006). In short, the bone of the skull was thinned out at the chosen location and a fibre‐optic bundle connected to a light source was placed above the thinned out region with a distance of a few millimetres. The light source had a wavelength of 561 nm and a power of 400 mW. The light source was turned on for 20 min and the dye Rose Bengal (1.3 mg/100 g body weight) dissolved in saline (9 mg NaCl/ml) (total volume 1 ml) was injected through the left femoral vein during the first 2 min of illumination. During sham experiments the procedure was identical to the above except that the light source was never turned on.

### Morphological analysis

Most animals were killed within 1 h of completed electrophysiological recordings using an overdose of pentobarbital. Five of the animals were instead, under maintained general anaesthesia, transcardially perfused using 100 ml phosphate buffered saline (PBS) followed by 75 ml 4% paraformaldehyde (PFA) solution. The brains were removed and then postfixed in 4% PFA solution for 48–72 h and then stored in PBS. Before sectioning the brain was submerged in 25% sucrose in PBS for 48 h. Thirty micrometre sections were then cut using a cryostat‐microtome and the sections were stained using a primary antibody against the neuron‐specific nuclear protein NeuN followed by a secondary antibody conjugated with Alexa Fluor 488. The sections were then studied using a confocal microscope and the extent of the stroke was measured using the software ImageJ (Rasband WS. ImageJ, US National Institutes of Health, Bethseda, MD, USA, imagej.nih.gov/ij, 1997–2012). A brain region was classified as lesioned by the induced stroke if the number of intact nuclei drastically decreased compared to surrounding tissue at the same cortical layer and the layers adjacent to it, as judged by visual inspection. The effect was confirmed by studying several adjacent sections. Note that within the lesioned brain region there was a mixture of more densely and less densely affected tissue, which likely correspond to the core infarct region and the penumbra zone (Hoff *et al*. 2005), respectively, to the extent that these areas can be differentiated already approximately 1 h after the stroke induction.

### Decoding of neuronal responses using principal component analysis

To determine to what extent the individual responses of the neurons could be used to identify the stimulation pattern applied to the skin of the second digit, we used a slightly modified version of a previously published method (Oddo *et al*. 2017). The method is based on the statistical methods of bootstrapping principal component analysis (PCA) and k‐nearest neighbour (kNN) classification. The rationale for the choice of methods is that due to biological noise arising from uncontrollable variations in the intrinsic brain network activity that could coincide in time with the provided stimulation, the neurons will respond differently to repeated applications of an input even if it generates identical patterns of primary afferent activation in the skin. In order to quantify the quality of the cortical information processing, we needed to quantify if the variability between the responses generated by repeated applications of the same stimulation pattern still permitted the evoked responses to be separated from those evoked by the other spatiotemporal input patterns. Hence, the measure of the information processing can be changed from the mere binary question, if the responses on average changed or not, to a more highly resolvable probability by which the responses evoked by the different stimulation patterns could be separated from each other. In short, bootstrapped responses were decomposed into their scalar products of the corresponding principal components (PCs) and thus positioned in a high‐dimensional PC space within which kNN‐classification could be applied to calculate this probability. As an internal control, we performed each analysis a second time, but now the labels of the stimulation patterns that evoked the responses were shuffled between the responses. The method is described in detail below.

(i) We converted the spike trains of each evoked response into continuous functions by convolving them using an exponential kernel with a characteristic time of 5 ms. The spike responses always included the first 1000 ms of the evoked responses.
(ii) We randomly assigned half of the convolved responses to a training set and the remaining half to a test set. Each set was handled separately for the remaining steps of the analysis.
(iii) We first used bootstrapping to resample the training data 200 times without stimulation labels (see below for further details). These bootstrapped data were merely used to calculate how many (*N*) principal components (PCs) were required to explain 95% of the variance.
(iv) Then, we used bootstrapping to resample the training and test set separately and computed the scalar product between each bootstrapped response and each of the *N* PC‐vectors using the least squares method. This was hence the process that positioned each bootstrapped response in the *N*‐dimensional space.
(v) Finally, we used the kNN‐classification procedure to decode the stimulation patterns from each bootstrapped response. For each bootstrapped response in the test set, the nine closest responses (nearest neighbours) in the training set were identified by Euclidean distance calculation in the *N*‐dimensional space. The response was classified as elicited by the same stimulation pattern that elicited the relative majority of these neighbours. This classification, or prediction, could therefore be correct or incorrect (relative to the actual stimulation pattern that evoked the response). Hence, for each stimulation pattern and condition, the percentage of the responses that were correctly classified, and at which percentages the misclassified responses were mistakenly indicated to belong to other classes, were obtained and expressed as a confusion matrix.
(vi) We performed 50 iterations of this decoding procedure, each with a different random division of the evoked responses into a training and a test set. The confusion matrix that we used for subsequent analysis and display was the average confusion matrix obtained from these 50 iterations.

The analysis above for each neuron generated a resulting confusion matrix that included 16 different classes (i.e. the eight different stimulation patterns under two conditions, before and after stroke). Note that such a confusion matrix is not symmetric, i.e. the combination S10_predicted_→S5_actual_ is different from S5_predicted_→S10_actual_. Furthermore, to estimate the decoding performance for each class we calculated two metrics, recall and precision. Recall (also known as Sensitivity) is the proportion of responses evoked by a specific stimulation pattern that was classified as belonging to that stimulation pattern. Precision is the proportion of responses classified as belonging to a specific stimulation pattern that actually belonged to that class. The harmonic mean of these two metrics is called the F‐score, which we use as the metric for the decoding performance, and which hence represents our high resolution measure of the cortical information processing.

#### Bootstrapping of time‐continuous signals

The convolved responses from each neuronal recording were bootstrapped by first grouping them by stimulation pattern. A new sample of *N* responses was taken from this population, where *N* equals the number of available responses, using sampling with replacement. The sum of these samples was stored as a bootstrapped response. It is important to note that sampling with replacement is not equal to the overall mean of the available responses. This separates this approach from PSTH‐based methods where only the overall mean is analysed and in‐group variability is averaged out.

#### Shuffled control decoding

The theoretical chance level of the decoding performance when performing a kNN‐classification analysis depends on the number of classes. The chance level for correct classification when there are two classes is 50%, i.e. 100% divided by the number of classes. In our setting, with sixteen classes, i.e. eight spatiotemporal tactile stimulation patterns under two conditions (pre‐ and post‐stroke), the theoretical chance level was hence 6.25%. For each neuron, we also tested the effect of shuffling the responses with respect to the stimulation pattern. This test was done to provide a control for each neuron, i.e. if there were features in the neuronal firing patterns, or in the method, that would bias neurons to not report chance decoding level when the generated responses were independent of the stimulation pattern. This control decoding analysis (referred to as ‘shuffled control decoding’ in the Results) worked exactly as the decoding analysis described previously, but before the data were split into a test and training set, the labels for each stimulation pattern were shuffled between the pool of recorded responses.

### Analysis of ECoG frequency bands and overall spike firing

The ECoG spectrogram for the 0–200 Hz frequency range was calculated for the entire duration of the experiment. Visual inspection of the spectrograms showed that changes in EEG power following stroke was particularly clear in the 30–100 Hz range. Therefore the mean power (dB) of the gamma‐band (30–100 Hz) was extracted for further analysis. The pre‐stroke signal was down‐sampled to approximately 0.1 Hz by means of smoothing with a moving average; subsequently the mean (baseline) and standard deviation were calculated. A significant deviation from baseline activity was defined as five consecutive bins of 10 s of at least two standard deviations below baseline. For each significant deviation in ECoG signal the closest significant deviation of spiking frequency was calculated, limited to deviations occurring within ±120 s of the ECoG deviation.

Sorted spike times were analysed in a similar manner. The spike times were down‐sampled to 0.1 Hz bins. From these bins, the pre‐stroke mean (baseline) and the standard deviation of the spiking activity was calculated. A significant deviation from baseline was defined to have occurred when there were at least three consecutive bins of at least two standard deviations below baseline activity.

The overall spike firing frequency was simply obtained by taking the number of spikes of the time continuous recording divided by the recording time. The coefficiency of variation (CV) was calculated as the standard deviation of all interspike intervals divided by the mean interspike interval. Both the frequency and the CV were calculated separately for each neuron and condition (pre‐stroke and post‐stroke, respectively).

### Brain state segmentation

For the analysis of the time spent in the desynchronized *versus* the synchronized brain state, the spectral density of the ECoG was calculated with a segment length of 1000 ms, an overlap of 125 ms and a constant (mean) detrending. The spectral density of delta, theta and alpha bands (0–12 Hz) was summed for each segment and the compound value was used for the remainder of the analysis. An asynchronous segment of ECoG was assumed to occur when the compound spectral density dropped below the compound spectral density median for at least two segments in sequence.

### Statistical analysis

The Kolmogorov–Smirnov test was used on the sets of mean decoding performance for pre‐ and post‐stroke conditions (as well as for pre‐ and post‐sham conditions) and firing frequency in order to test if they were normally distributed. According to the test, none of the data sets were normally distributed and further analysis was thus made using non‐parametric tests. When comparing the stroke and sham conditions regarding the net difference in F‐score between pre‐ and post‐stroke/sham, the data were found to be normally distributed when studying the marginal histograms (Fig. 4). This was further verified by studying the respective quantile–quantile (Q–Q) plots and thus a parametric test was used in this comparison.

In order to evaluate if there was any difference between the pre‐ and post‐conditions with respect to decoding performance or mean firing frequency, a paired two‐sided Wilcoxon signed rank test was used. A linear regression model was used to see if any trends could be observed when comparing pre‐stroke decoding performance with change induced by stroke, and pre‐stroke firing frequency and post‐stroke change in firing frequency. In order to evaluate the fit of the linear models, their residuals were studied both as histograms and Q–Q plots, which both indicated that the residuals were normally distributed, and as scatter plots, plotted against their respective explanatory variable, in which no trends could be detected.

## Results

**Figure 1:**
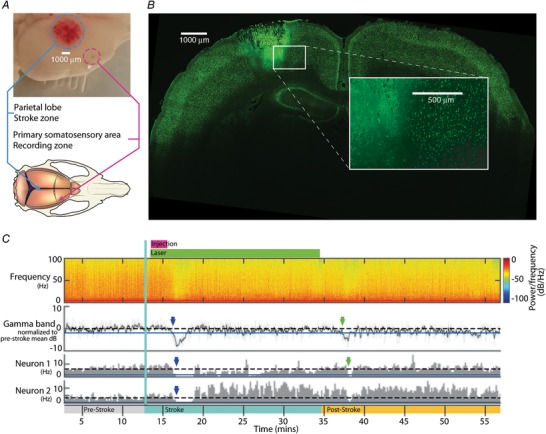
Extent of photothrombotic lesions and effects on spontaneous cortical neural activity *A*, top: photograph of a brain with stroke lesion in the parietal surface of the neocortex; bottom: illustration of the rat brain and skull. The recording and stroke sites are indicated in both images. *B*, transversal histological section of the brain including a stroke lesion area. NeuN staining. *C*, spectral analysis of the ECoG recorded near S1 in parallel with two S1 single cell spike recordings. The duration of the pre‐stroke, stroke and post‐stroke protocols are indicated by the coloured segments of the time axis. The onset of the photothrombotic lesion protocol is also indicated by the vertical green line. The occurrences of spreading depression (SD) in the ECoG, as detected by suppressions in the gamma frequency band (here including 30–100 Hz), is indicated by the downward arrows. Note the parallel dramatic decrease in the neuronal spike firing activity, as indicated by corresponding arrows in the time histograms of the neuronal spike firing. Dashed horizontal lines indicate the pre‐stroke mean activity, blue continuous horizontal lines (close to 0 Hz for Neuron 2) indicate a deviation of 2 standard deviations from the mean. [Color figure can be viewed at http://wileyonlinelibrary.com]

In order to quantify the information processing deficits induced by localized cortical lesions, we combined an analysis of the decoding performance of tactile input patterns in neocortical neurons with localized photothrombotic, stroke‐like lesions in a separate part of the cortex with a parietal location (Fig. 1 *A*). Inclusion in the material required the neuronal recordings to last for at least 62 min, the minimum period required to obtain a full set of control responses (20 min), keeping the neuron during the stroke induction (22 min) and thereafter obtaining a full set of conditioned responses (20 min) to quantify the resulting impairment in the neuronal decoding of the pre‐set tactile input patterns. The pause between the end of the stroke induction and the recording of the conditioned responses was in the order of 1 min. In 12 experiments, we managed to obtain sufficiently long‐term recordings from a total of 22 neurons (double electrode recordings were successful in most experiments) located in the paw area of the right S1 (contralateral to the paw stimulation) of the neocortex, where an injection of Rose Bengal was combined with a focal light beam to induce a local lesion. In addition, 10 neurons were recorded in 10 sham experiments where the same procedure was repeated, with Rose Bengal injection and all other preparations but without turning on the power for the focal light beam. The neurons were recorded at depths ranging from 440 to 1246 µm from the cortical surface, using one or two separate patch clamp electrodes mounted on different micromanipulators.

### Morphological and general physiological effects of photothrombotic stroke

We first estimated the tissue volume affected by the standardized photothrombotic lesioning protocol at the macroscopic (Fig. 1 *A*) and microscopic level (Fig. 1 *B*) in five of the brains post‐mortem. For the ‘trapped’ Rose Bengal in the thrombotic tissue (Fig. 1 *A*, top) the maximal extent in the rostrocaudal plane was 3.8 ± 0.3 mm (mean ± standard deviation) (95% confidence interval (CI) 3.31–4.08 mm) and the mean mediolateral extent was 3.5 ± 0.3 mm (CI 3.03–3.97 mm). In histological analysis (Fig. 1 *B*), the mean extent of the stroke in the rostrocaudal axis was estimated to be 4.1 ± 1.0 mm (CI 2.87–5.34 mm), to 3.8 ± 1.0 mm (CI 2.59–4.99 mm) in the mediolateral axis and 2.6 ± 0.2 mm (CI 2.31–2.89 mm) in the dorsoventral axis (depth). The distance between the centre points of the stroke region and of the recording region was estimated to 6.2 ± 0.45 mm. Hence, based on the measured diameter of the stroke area, the distance from the outer limit of the stroke region to the recording area was in the order of 3.0–3.5 mm. To evaluate the acute physiological effects of the induced stroke, we recorded the spiking frequency of the neurons and the ECoG continuously (Fig. 1 *C*). Stroke induction typically resulted in a temporary, delayed but sudden decrease in ECoG activity that was often paralleled by a temporary decrease in neuronal spiking activity, i.e. spreading depression (SD) as known to occur in stroke (Strong *et al*. 2002; Lauritzen *et al*. 2011). The decrease in ECoG activity was found to be most prominent in the gamma frequency band (30 –100 Hz). The median onset latency time for the significant decrease in the gamma band activity, relative to the start of the stroke induction protocol, was 321 s, whereas the mean of the onset latency time was 275 s. The drop in ECoG activity was typically followed by a significant temporary drop in neuronal spiking frequency with a median absolute latency time difference of 34 s and a mean absolute difference of 25 s. According to this definition, 8 out of 12 experiments exhibited a parallel drop in ECoG and spiking activity.

### Quantification of stroke effects on neuronal decoding

**Figure 2:**
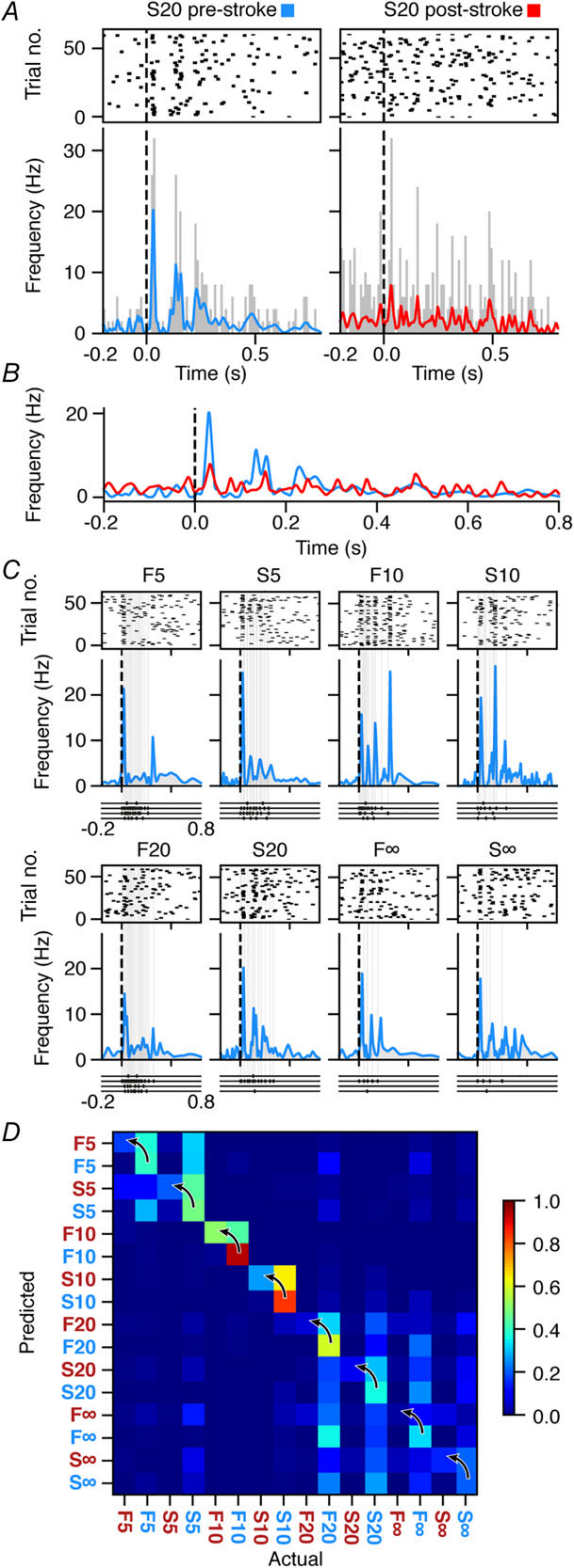
Neuronal responses and decoding of an S1 neuron before and after stroke *A*, raster plots and peristimulus time histograms (PSTHs) of the spiking activity relative to the onset of the stimulation pattern. The PSTHs are overlaid with a kernel density estimation (KDE) of the spike responses. It is the responses that were evoked by the ‘S20’ stimulation pattern before and after stroke that are shown. *B*, overlay of the KDEs of the evoked responses pre‐ and post‐stroke. Dashed line indicates stimulus onset. *C*, raster plots and KDEs of the responses evoked by each of the eight spatiotemporal stimulation patterns before stroke. The rasters below each panel indicate the times of stimulation of each of the four skin sites for each of the stimulation patterns used (with the corresponding labels shown at the top of each panel). Extended vertical dashed line indicates these times of stimulation in the response plots. *D*, confusion matrix of the decoding performance (calculated as the F‐score) of the sample neuron across all eight stimulation patterns before (blue labels) and after stroke (red labels). The arrows indicate the elements in the confusion matrix that compare the effect of stroke for the same stimulation patterns. [Color figure can be viewed at http://wileyonlinelibrary.com]

To quantify the information processing in the cortical neurons, we used a previously introduced method (Oddo *et al*. 2017) designed to obtain maximally reproducible spatiotemporal patterns of tactile afferent input to the brain. The reproducibility of the input is important to minimize the variability of the input information to be processed and thereby allows for a high resolution quantification of the neuronal decoding. The decoding performance of the neuron is in turn an indicator of the integrity of the neuronal network that the recorded neuron is situated in. We used exactly the same set of eight predefined spatiotemporal patterns of electrical skin stimulation, distributed across four different skin sites, to the volar side of the distal contralateral digit 2. As the underlying process to generate these stimulation patterns are not important here, we refer the interested reader to the paper where the whole process is described in detail (Oddo *et al*. 2017). The spatiotemporal patterns used are shown underneath the relevant graphs of the responses shown in Fig. 2.

Figure 2 *A* illustrates the spike responses to one of the eight stimulation patterns before and after stroke in raster plots, peristimulus time histograms (PSTHs) and kernel density estimations (KDEs). Following the lesion, even though the overall response structure seemed preserved, the sequential response peaks and troughs that defined the shape of the response could be quite dramatically reduced (Fig. 2 *B*). This effect can be expected to make it much harder to use the spike responses of the neuron to differentiate one input pattern from another, i.e. the informational value of the output of the neuron becomes impaired. To quantify the effect, we analysed the decoding performance of each neuron for all of the eight spatiotemporal input patterns under the two different conditions (pre‐ and post‐stroke) (Fig. 2 *C*). In the example neuron, the stroke lesion resulted in a clear reduction of the mean decoding performance and this reduction was seen for almost every single input pattern (Fig. 2 *D*).

**Figure 3:**
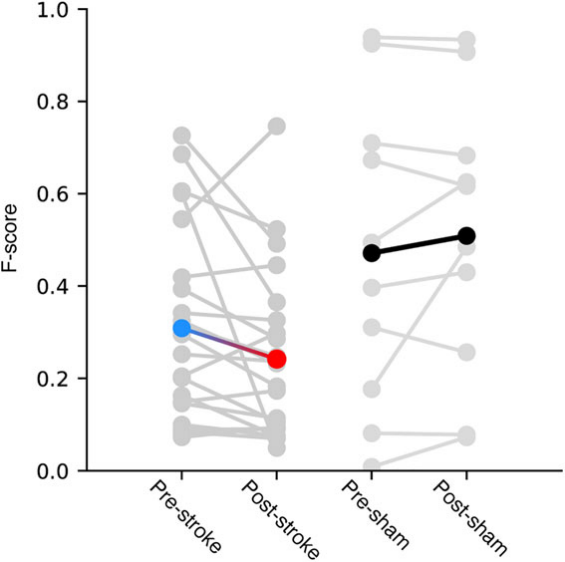
Effects of stroke lesion on the mean decoding performance of S1 neurons To the left, mean decoding performance for all individual S1 neurons before and after stroke. The blue–red line represents the mean of all neurons. To the right, similar display but for the sham experiments. [Color figure can be viewed at http://wileyonlinelibrary.com]

The mean decoding performance for the population of neurons pre‐stroke was 30.9% (*n* = 22, median = 27.4%, interquartile range (IQR) = 26.6%; shuffled control: mean = 8.0%, median = 8.5%, IQR = 3.2%), and post‐stroke 24.2% (*n* = 22, median = 20.8%, IQR = 22.5%; shuffled control: mean = 7.6%, median = 6.9%, IQR = 2.7%). Furthermore, the mean decoding performance for the population of neurons pre‐sham was 47.1% (*n* = 10, median = 44.5%, IQR = 49.0%; shuffled control: mean = 6.2%, median = 6.1%, IQR = 0.5%), and post‐sham 50.9% (*n* = 10, median = 55.1%, IQR = 36.8%; shuffled control: mean = 6.0%, median = 5.9%, IQR = 0.6%). Although the decoding actually markedly increased in 3/22 cases, a reduction of the mean decoding performance was a statistically significant effect of parietal stroke across the population of S1 neurons (Fig. 3; two‐sided Wilcoxon signed rank test, degrees of freedom (d.f.) = 21, *P* = 0.028, α level *P* < 0.05, rank sum = 59.0). In contrast, no statistically significant difference was found in the population of neurons when they were recorded before and after the sham stroke condition (Fig. 3; two‐sided Wilcoxon signed rank test, d.f. = 9, *P* = 0.64).

**Figure 4:**
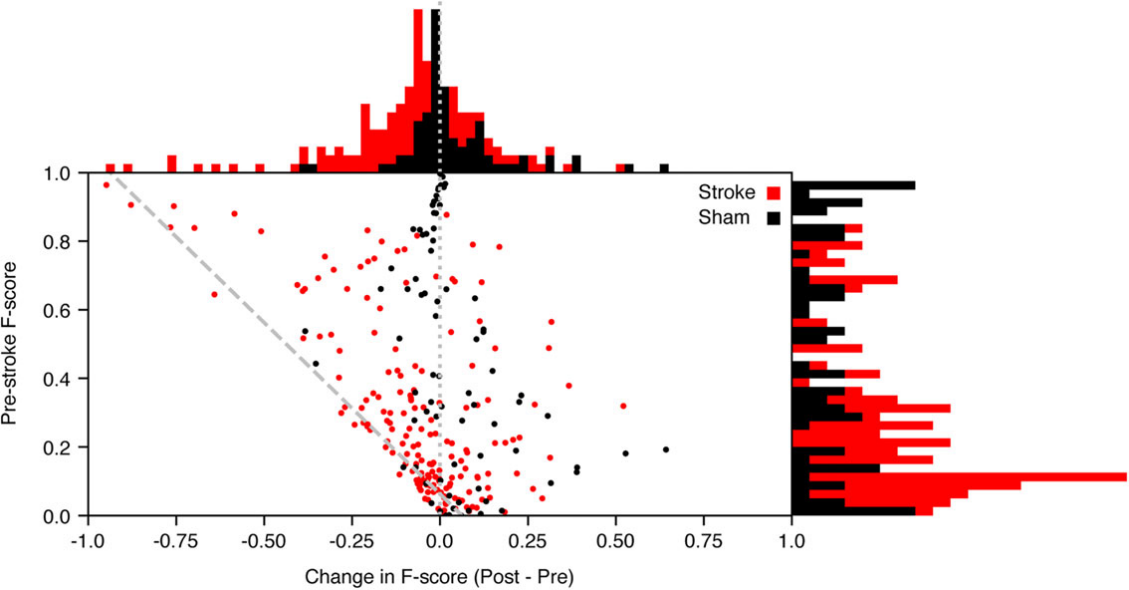
Net change in decoding performance post‐stroke as a function of decoding performance pre‐stroke for each stimulation pattern and neuron The data are shown as a scatter plot and as frequency distribution histograms. In the scatter plot, the dashed diagonal line indicates the change in decoding required to reach chance level decoding for each given pre‐stroke decoding performance level whereas the dotted vertical line indicates zero change. [Color figure can be viewed at http://wileyonlinelibrary.com]

Although the effects of stroke on the mean decoding performance at the population level were statistically significant, they were often not dramatic. We observed, though, that for individual stimulation patterns that were initially decoded with a high certainty, the distant stroke could cause a major drop in decoding performance. To illustrate this, we plotted the decoding of individual stimulation patterns, across all neurons, against their net change after stroke (Fig. 4). A remarkable difference between stroke and sham conditions was that major stroke‐induced reductions in decoding performance occurred only for input patterns that initially were decoded with high precision (high F‐score). As shown by the diagonal line in Fig. 4, which illustrates how close to chance level the reduction of the decoding performance fell post‐stroke, a dramatic reduction could theoretically only occur for patterns that were initially decoded with a high precision. In fact, for input patterns with very low pre‐stroke decoding, in some cases even decoding below chance, stroke could even result in an increase of the decoding performance, indicating that decoding close to chance level was not sensitive to stroke. In contrast, inputs with initially high decoding levels, which presumably depended on a more well‐organized information flow through the network, were much more vulnerable to stroke. Nevertheless, the differences between stroke and sham conditions in terms of net reduction in decoding performance were statistically significant and independent of the minimal threshold level of control decoding performance used as an inclusion criteria for the statistical analysis (Student's *t* test comparing complete population during stroke condition against sham condition, *t* statistic = −3.98, *P* = 8.94 × 10^−5^). The theoretically possible magnitude of the change (as indicated by the diagonal line in Fig. 4) could imply that the difference seen across the whole population was artificial, i.e. the low decoders could only move to a higher decoding while medium to high decoders could move in either direction. To verify our result, we thus tested if an arbitrary exclusion cutoff would alter the outcome. We iteratively remade the *t* test and excluded data with a pre‐stroke F‐score less than an increasing cutoff (range = 0.00–1.00, increase = 0.005). No cutoff resulted in a *P* value above the α level of 0.05, which would imply that our initial result is valid. As a separate, unrelated issue, it can be noted that we have previously found that there is no relationship between decoding performance and the neuronal depth in the S1 cortex for the same set of tactile inputs (Oddo *et al*. 2017), but the number of neurons in the present study was too low to allow a proper analysis of the issue.

### Stroke caused no systematic differences in average neuronal firing frequency or global brain state

**Figure 5:**
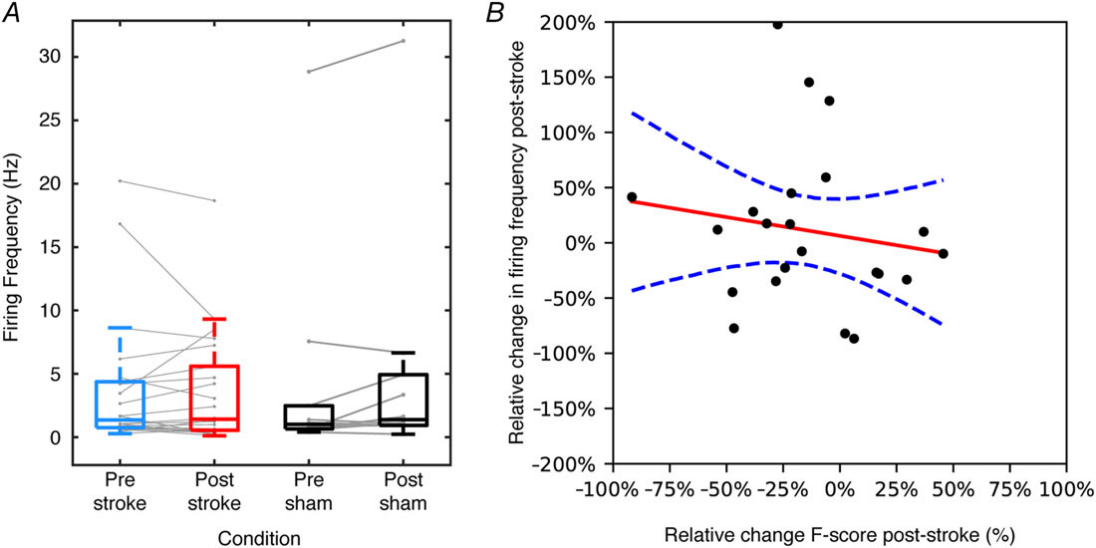
Relationship between changes in firing frequency and in decoding performance following stroke *A*, the firing frequency of each neuron before and after stroke with an overlaid box plot. The corresponding data for the sham stroke is shown to the right. *B*, for each neuron, the relationship between the relative change in firing frequency and net change in mean decoding performance after stroke. The panel also illustrates a linear regression and its 95% confidence interval (*R* ^2^ = 2.3%, *P* = 0.499, intercept = 0.63, slope = −0.339; CI 95% = −1.149 to 0.471). [Color figure can be viewed at http://wileyonlinelibrary.com]

One factor that could have at least partly explained the lesion‐induced impairment on the decoding performance would be if the average firing frequency of the neurons altered in a systematic fashion. However, no such systematic change of firing frequency was observed (Fig. 5 *A*; two‐sided Wilcoxon signed rank test (*P* = 0.9224, α level *P* < 0.05; for the sham experiments there was no statistically significant change either, *P* = 0.2334). We also tested whether the changes in firing frequency that occurred might have affected the decoding performance with a certain bias depending on the polarity of the change in firing frequency (increase *vs*. decrease). No such statistically significant relationship was found according to a linear regression analysis (Fig. 5 *B*; *n* = 22, coefficient of determination (*R* ^2^) = 2.3%, *P* = 0.499, intercept = 0.63, slope = −0.339; CI 95% = −1.149 to 0.471). While on average we found no changes in firing frequency, it is notable that some neurons halved their firing frequency whereas other nearly doubled it (Fig. 5 *A*). We therefore followed up this observation with a further analysis of the spike firing regularity, or its CV. This measure did change following stroke. Before stroke the CV was 1.29 ± 0.23, whereas after stroke the CV increased to 2.02 ± 1.36, a change that was statistically significant (*P* = 0.0001, two‐sided Wilcoxon signed rank test). Following sham stroke, no such change was observed (CV before sham stroke was 1.19 ± 0.42, after sham stroke it was 1.20 ± 0.46, *P* = 0.6953). The variable changes in firing frequency could be an indication of that when the global neocortical network structure is perturbed, the balance of activation in the network shifts in many subcircuits, so that some neurons may become less excitable and others more excitable. Whereas such changes in excitability may average out across the population, the spike firing regularity could instead be expected to change more unidirectionally. As spike firing regularity has been argued to be a measure of what forms of information are encoded by the neuron (Mochizuki *et al*. 2016), disruption of this parameter could be one explanation for the observed impairment of the information processing (Figs 2 and 3).

We made a further control using the ECoG. In ketamine–xylazine anaesthesia, the brain EEG state fluctuates between desynchronized and synchronized activity (Chauvette *et al*. 2011). If the ratio of the time spent in the synchronized *versus* the desynchronized condition alters after stroke, this could potentially explain a degradation of the decoding performance as we have previously observed that the decoding performance is somewhat lower in the synchronized state under similar experimental conditions to those in the present study (Enander *et al*. 2019). We found that the mean time spent in the asynchronous ECoG state prior to stroke was 23.2% (±2.1%, *n* = 12) of the total recording time, and for post‐stroke the mean was 23.6% (±1.8%, *n* = 12). A dependent *t* test was performed, and no statistical difference could be found (*P* = 0.555, *n* = 12). For the sham data, the corresponding numbers were 25.9% (±5.3, *n* = 5) pre‐stroke and 27.8% (±6.0%, *n* = 5) post‐stroke, again with no statistical difference (Student's dependent *t* test, *P* = 0.662). Hence, stroke did not alter the fraction of time the brain was in the desynchronized ECoG state.

## Discussion

Our analysis shows that local information processing in the neocortex, expressed as the neuronal decoding performance across eight tactile input patterns delivered to the same small part of the second digit, is consistently impaired after distant stroke‐like lesions. This finding indicates that even the primary neocortical processing of tactile information depends on the integration of input from widespread neocortical circuitry. Thereby, it suggests that neocortical processing is far from being strictly modularized and offers an explanation for the growing clinical realization that the deficits following local lesions are often not conforming to the classical belief of a localized functional specificity.

### What is shown by the decoding analysis

Skin–object interactions result in spatiotemporal patterns of primary afferent activation (Jörntell *et al*. 2014) that via divergent connections in turn generate spatiotemporal patterns of cuneate, thalamic and neocortical neuronal activation. The accumulated lifetime experiences are likely to contribute to the physiological structure of the neuronal network, which in turn shapes the way the afferent information is routed between the neurons. This network can be assumed to involve multiple excitatory and inhibitory neurons (Oddo *et al*. 2017) whose activity needs to be sufficiently balanced to propagate the information through the neocortical network (Pei *et al*. 2011) without leading to positive feedback loops of recurrent excitation. In addition, ‘top‐down’ influences from within the cortex can be expected to contribute to this routing and in principle these are all the components the information processing is composed of. What we read out with the decoding analysis is with what precision the spike output activity of a single node of that network can be used to separate the eight input patterns we provided. As we have previously found, within the S1 paw region all neurons recorded carry information about these input patterns (Oddo *et al*. 2017), and naturally likely many more patterns. The effects we obtained after the remote focal cortical lesion indicate that the network required to produce the information processing we hence observed in S1 is dependent on the integrity of the network far away from the recording site. This dependency could be due to the core processing network extending all the way to the remote site, and therefore most likely to many other remote parts of the cortex, or that the remote site somehow provides a permissive excitation for the S1 local circuitry, in which case it will still affect S1 processing. It could in principle also depend on the remote site affecting the excitability of the cuneate–thalamic transmission of the primary afferent input. Indeed, there are multiple anatomical substrates for such network operations, such as horizontal cortico‐cortical connections or cortico‐thalamo‐cortical connections (Frostig *et al*. 2008; Negyessy *et al*. 2013; Ashaber *et al*. 2014; Sherman, 2016; Gerfen *et al*. 2018).

### Properties of the approach to generate the analysed responses

Because of the high reproducibility of the input patterns across all repetitions, as well as their high resolvability (Oddo *et al*. 2017), we believe that our approach is one of the most sensitive means published to measure functional neuronal network integrity globally in the neocortex. It can hence be expected to detect effects of localized lesions with a higher resolution than other methods have allowed so far. The reproducibility achieved through the electrical stimulation of tactile afferents is impossible to achieve using mechanical stimulation (Hayward *et al*. 2014), but has the drawback that it is at risk of being outside the range of normal spatiotemporal activation patterns. However, the patterns used were previously generated using a bionic fingertip prosthesis during simulated active touch, and have several properties in common with the natural activation patterns of slowly and rapidly activating tactile skin afferents, such as their overall firing frequency and the temporal envelope of the firing in the individual tactile afferents being activated (Jenmalm *et al*. 2003; Oddo *et al*. 2017). Even factoring in the potential level of synchrony at the level of the tactile afferent activation at each respective skin site/input channel, the tactile afferent input patterns used are biologically feasible (Oddo *et al*. 2017). The neuronal spike responses generated by the input patterns are likely to involve up to 100s of synaptic relays in cuneate, thalamus and neocortex and are thus bound to reflect the natural network function to some degree.

### Changing views on the organization of cortical processing

It is gradually becoming clear that the neocortex is a globally interconnected structure and that a strict idea of functional localization is becoming less tenable. For example, it was recently shown that tactile processing in principle could occur anywhere within the neocortex, including the primary visual cortex (Enander *et al*. 2019). Already Arbib *et al*. (1998) estimated that any neuron in the neocortex connects to any other neocortical neuron with synaptic linkages involving no more than five neurons on average. Recent neuroanatomical evidence suggests that the neocortex may be even more tightly interconnected. Cortico‐cortical connections to and from each single cortical neuron can be incredibly widespread (Wall *et al*. 2016; Gerfen *et al*. 2018; Lin *et al*. 2018; Economo *et al*. 2019). Moreover, activity generated in one part of the cortex can in principle reach any other part of the cortex via a single synaptic relay in the thalamus (Sherman, 2016). Hence, whereas the present study focused on the degrading effects a stroke with a parietal location had on S1 processing, because our methodology depended on the analysis of responses to tactile responses with a very high degree of reproducibility, there is reason to expect that an inter‐area functional dependency of the type described here will be found to apply to the interactions between any two areas of the cortex. Future studies that find alternative methods to make as precise quantification of the information processing as the present methodology will be required to confirm this prediction.

### Spreading depression

Following stroke induction, we could observe spreading depression (SD) in both the ECoG and neuronal firing (Fig. 1 *C*). SD is characterized by a self‐propagating, synchronous, wave‐like depolarization of neurons and glia, spreading out from a point of origin (Strong *et al*. 2002; Sawant‐Pokam *et al*. 2017). A geographic spread over time matches our observations of somewhat delayed effects at the recording sites, being at a distant location from the stroke lesion site, and of a delay in timing between the effects being observed in the ECoG and in the neuronal spiking. SD can be regarded as a pathophysiological reaction in the neuronal networks that are functionally connected to the site of the lesion. All parts of the cortical network that are being functionally interconnected with the lesion site can therefore be affected. Depending on the degree of interconnectivity, this region can become very large, possibly encompassing all parts of the neocortex. The fact that we could record SD in the neurons within S1 corroborates the notion that the degree of functional interconnectivity in the brain is very high.

### Relationship to deficits observed in stroke patients

Hence, both the decoding analysis of the neuronal spike responses and the SD suggest that there are widespread functional dependencies between different parts of the neocortex. Whereas a previous study found this to apply to between‐hemispheres control of inhibitory interneurons (Mohajerani *et al*. 2011), here we instead focused on within‐hemisphere effects on our high‐resolution measure of neuronal information processing. Apart from our results, distributed network processing is also suggested by EEG recordings in humans processing tactile input (Genna *et al*. 2017). Indeed, parietal cortical stroke in man is almost always found to lead to deficits in the tactile sensory modality when a sufficiently careful examination is made (Bassetti *et al*. 1993; Connell *et al*. 2008) and is even associated with tactile sensory deficits additionally in the ipsilateral hand (Brasil‐Neto & de Lima, 2008). Naturally, we cannot directly address the question of exactly how the widespread impairments following a local lesion would translate to perceptual deficits or the exact role of the information from all different cortical regions under normal behaviour. This should be the aim of future studies. However, our findings do indicate that a vast variety of functional deficits, which in the clinical investigation will sometimes be suprathreshold and sometimes subthreshold to the specific performance indicator used, are to be expected in stroke patients.

### Conclusions

The present results show that focal stroke‐like lesions in the neocortex can affect neuronal information processing at distant locations from the lesion site. Since the neuronal information processing is dependent on the information it receives from other neurons, this finding suggests that the function of the neocortex depends on neurons that are globally interconnected. Specifically, we found that parietal cortical lesions affected the decoding of tactile input patterns in neurons of the S1 cortex. This further indicates that even the primary processing of sensory inputs is subject to control from remote cortical areas. For stroke patients, this finding provides an explanation for commonly observed widespread functional deficits following localized strokes.

## Additional information

### Competing interests

The authors declare no competing interests.

### Author contributions

A.W., J.M.D.E., F.B. and H.J. designed the lesion model. A.W. and J.M.D.E. performed the recording experiments and analysed the physiological data. All authors drafted the paper and A.W., J.M.D.E. and H.J. finalised it. All authors approved the final version of the manuscript and agree to be accountable for all aspects of the work in ensuring that questions related to the accuracy or integrity of any part of the work are appropriately investigated and resolved. All persons designated as authors qualify for authorship, and all those who qualify for authorship are listed.

### Funding

This work was supported by Hjärnfonden, the EU Grant FET 829186 ph‐coding (Predictive Haptic COding Devices In Next Generation interfaces), the Swedish Research Council (project grant no. K2014‐63X‐14780‐12‐3) and the Italian Ministry of Foreign Affairs and International Cooperation, Directorate General for Country Promotion (Economy, Culture and Science) – Unit for Scientific and Technological Cooperation and the Swedish Research Council, via the Italy–Sweden bilateral research project J52I15000030005 SensBrain (Brain network mechanisms for integration of natural tactile input patterns).
